# Effects of Digital Physical Health Exercises on Musculoskeletal Diseases: Systematic Review With Best-Evidence Synthesis

**DOI:** 10.2196/50616

**Published:** 2024-01-23

**Authors:** Johanna Nagel, Florian Wegener, Casper Grim, Matthias Wilhelm Hoppe

**Affiliations:** 1 Movement and Training Science Leipzig University Leipzig Germany; 2 Center for Musculoskeletal Surgery Osnabrück Klinikum Osnabrück Osnabrück Germany

**Keywords:** mobile health, mHealth, electronic health, eHealth, digital health applications, DiGA, musculoskeletal, MSK, home-based, PROM, disorder, mobile phone

## Abstract

**Background:**

Musculoskeletal diseases affect 1.71 billion people worldwide, impose a high biopsychosocial burden on patients, and are associated with high economic costs. The use of digital health interventions is a promising cost-saving approach for the treatment of musculoskeletal diseases. As physical exercise is the best clinical practice in the treatment of musculoskeletal diseases, digital health interventions that provide physical exercises could have a highly positive impact on musculoskeletal diseases, but evidence is lacking.

**Objective:**

This systematic review aims to evaluate the impact of digital physical health exercises on patients with musculoskeletal diseases concerning the localization of the musculoskeletal disease, patient-reported outcomes, and medical treatment types.

**Methods:**

We performed systematic literature research using the PRISMA (Preferred Reporting Items for Systematic Reviews and Meta-Analyses) guidelines. The search was conducted using the PubMed, BISp, Cochrane Library, and Web of Science databases. The Scottish Intercollegiate Guidelines Network checklist was used to assess the quality of the included original studies. To determine the evidence and direction of the impact of digital physical health exercises, a best-evidence synthesis was conducted, whereby only studies with at least acceptable methodological quality were included for validity purposes.

**Results:**

A total of 8988 studies were screened, of which 30 (0.33%) randomized controlled trials met the inclusion criteria. Of these, 16 studies (53%) were of acceptable or high quality; they included 1840 patients (1008/1643, 61.35% female; 3 studies including 197 patients did not report gender distribution) with various musculoskeletal diseases. A total of 3 different intervention types (app-based interventions, internet-based exercises, and telerehabilitation) were used to deliver digital physical health exercises. Strong evidence was found for the positive impact of digital physical health exercises on musculoskeletal diseases located in the back. Moderate evidence was found for diseases located in the shoulder and hip, whereas evidence for the entire body was limited. Conflicting evidence was found for diseases located in the knee and hand. For patient-reported outcomes, strong evidence was found for impairment and quality of life. Conflicting evidence was found for pain and function. Regarding the medical treatment type, conflicting evidence was found for operative and conservative therapies.

**Conclusions:**

Strong to moderate evidence was found for a positive impact on musculoskeletal diseases located in the back, shoulder, and hip and on the patient-reported outcomes of impairment and quality of life. Thus, digital physical health exercises could have a positive effect on a variety of symptoms of musculoskeletal diseases.

## Introduction

### Background

A total of 1.71 billion people are affected by musculoskeletal diseases worldwide [1]. They are characterized by chronic pain, functional disability, impairment, and reduced quality of life [1,2]. The most commonly affected body regions are the lower back and neck, with a period prevalence over the last 12 months of up to 61.3% and 45.7% [3], respectively, and a common disease is osteoarthritis, with a prevalence of up to 17.9% [4]. In addition to the high biopsychosocial burden [5], the evident increase in the incidence of musculoskeletal diseases over the last decades [6] results in high economic costs because of lost workdays and conservative or operative medical treatments [5]. To overcome such undesirable consequences, evidence-based, effective, and cost-saving health interventions are required. Therefore, the use of digital health interventions is a promising approach.

Digital health interventions aim to manage a wide range of diseases and health issues using digital devices such as smartphones, tablets, computers, or wearables, including mobile apps, telerehabilitation and web-based physician visits, web-based interactive programs, or tracking tools [7]. The use of mobile apps is increasing, with common intervention types categorized as physical exercise and fitness, lifestyle and stress, diet and nutrition, or medication reminders and educational materials [7]. In some countries, such as Germany, so-called digital health applications are also supported by health insurers after being evaluated as medical devices [8]. However, owing to their cost-saving potential and the increasing number of commercially available digital health interventions [7], further research is needed to evaluate the impact of different types of digital health interventions on specific diseases.

Previous systematic reviews have extensively evaluated the impact of digital health interventions on internal diseases. Positive effects have been demonstrated in treating chronic obstructive pulmonary disease [9], cardiovascular disease [10], and diabetes [11]. These effects encompass improvements in clinically relevant outcomes such as quality of life, health-related impairments, amelioration of risk factors and their consequences, as well as the control and management of HbA1c levels. For musculoskeletal diseases, only 2 previous systematic reviews have evaluated the impact of digital health interventions as a primary outcome. One review [12] showed that there are substantial clinical benefits in the management of musculoskeletal diseases for the patient-reported outcomes of pain (9 out of 19 studies) and functional disability (10 out of 16 studies). The results show that digital health interventions as adjuncts and as stand-alone treatments are not inferior but partly superior compared with interventions based on standard therapy, nondigital self-management, noninteractive digital measures, or no intervention. However, in this previous review, no evidence synthesis was performed. In addition, a further review [13] conducted a meta-analysis and showed moderate-quality evidence that digital health interventions are effective in reducing pain and improving function and self-management in patients with musculoskeletal disease. The included studies considered digital health interventions as interventions that are to be used only at home and as adjuncts to standard clinical care, compared with standard care, noninteractive digital interventions, or no intervention. Taken together, the use of digital health interventions as an adjunct to regular therapy could have positive health-related effects for both internal and musculoskeletal diseases, although less evidence is available for the latter.

However, little is known about the relationship between clinically relevant factors, such as the localization of the musculoskeletal diseases, patient-reported outcomes, or the type of applied conservative or operative medical treatments, and the effects of different types of digital health interventions in the treatment of musculoskeletal diseases. In terms of evidence-based medicine, such relationships must first be clarified when using digital health interventions as a regular treatment option for specific musculoskeletal diseases. Because of the increasing number of original studies, more systematic research is needed to review and assess the existing evidence. Previous systematic reviews [12,13] have included all types of digital health interventions, providing a comprehensive overall result across all biopsychosocial domains. As physical exercise is the best clinical practice for the treatment of musculoskeletal diseases [14], digital physical health exercises could have a highly positive impact on musculoskeletal diseases. However, little is known about how the effects of digital physical health exercises are related to the aforementioned clinically relevant factors.

### Objective

Therefore, this systematic review aimed to evaluate the impact of digital physical health exercises on patients with musculoskeletal diseases concerning the localization of the musculoskeletal disease, patient-reported outcomes, and medical treatment types. In addition, a best-evidence synthesis was conducted to estimate the direction and strength of the existing evidence.

## Methods

### Research Design and Eligibility Criteria

The systematic review was conducted according to the PRISMA (Preferred Reporting Items for Systematic Reviews and Meta-Analyses) guidelines [15]. Eligibility criteria according to the population, intervention, comparison, outcome, study design (PICOS) scheme [16] were applied. Table 1 presents the inclusion criteria according to the PICOS scheme. Textbox 1 presents the search line.

The corresponding keywords are also presented. Studies were not reviewed if they did not report on a specific musculoskeletal disease, if the digital health intervention included no physical exercises, if no control group was considered, or if none of the included patient-reported outcomes were assessed as a primary outcome. All methodological steps were performed by 1 author and validated by a second author. Uncertainties were discussed until consensus was reached. Because of the literary nature of this study, ethics approval was not required.

**Table 1:** PICOS^a^ scheme for the definition of the inclusion criteria and the presentation of the corresponding keywords.

	Population	Intervention	Comparison	Outcome	Study design
Inclusion criteria^b^	Patients with any musculoskeletal disease according to the definition of the WHO^c^	Any digital health intervention using home-based physical exercises	Any conventional or no therapy	Patient-reported outcomes pain, function, disability, and quality of life assessed by established and validated clinical questionnaires or scales	Randomized controlled trials
Keywords	“Musculoskeletal disease” OR “Musculoskeletal disorder” OR “Musculoskeletal pain” OR “Chronic pain” OR “Acute pain” OR “Overuse pain” OR “Chronic injury” OR “Chronic injuries” OR “Acute injury” OR “Acute injuries” OR “Overuse injury” OR “Overuse injuries” OR “Chronic disease” OR “Acute disease” OR “Overuse disease” OR “Osteoporosis” OR “Osteoarthritis” OR “Rheumatoid arthritis” OR “Tendinopathy” OR “Tendinopathies” OR “Rotator cuff” OR “Lower extremity” OR “Lower extremities” OR “Upper extremity” OR “Upper extremities” OR “Hip” OR “Knee” OR “Foot” OR “Hand” OR “Ankle” OR “Wrist” OR “Elbow” OR “Low back” OR “Neck” OR “Back” OR “Spine” OR “Shoulder” OR “Arm” OR “Leg” OR “Muscle” OR “Tendon” OR “Ligament”	“Digital movement therapy” OR “Digital movement therapies” OR “Mobile health” OR “eTherapy” OR “eTherapies” OR “Web-based intervention” OR “Digital intervention” OR “Computer-based intervention” OR “App-based intervention” OR “Digital health application” OR “Technology-assisted therapy” OR “Technology-assisted therapies” OR “Internet-based intervention” OR “Computer-assisted therapy” OR “Computer-assisted therapies” OR “health app” OR “mobile application” OR “Smartphone” OR “Mobile phone” OR “ehealth” OR “mhealth” OR “telerehabilitation” OR “Telemedicine” OR “online intervention” OR “internet-delivered intervention”	“Osteopathy” OR “movement therapy” OR “movement therapies” OR “physical therapy” OR “physical therapies” OR “therapeutic exercise” OR “medical gymnastic” OR “traditional therapy” OR “traditional therapies” OR “manual therapy” OR “manual therapies” OR “physiotherapy” OR “No therapy” OR “No therapies” OR “conventional therapy” OR “conventional therapies” OR “no treatment” OR “no intervention” OR “watch-and-wait” OR “wait-and-see” OR “watch and wait” OR “wait and see”	N/A^d^	“Randomized controlled trials”

^a^PICOS: Population, intervention, comparison, outcome, and study design.

^b^Others: Studies in English or German language with free full access were included.

^c^WHO: World Health Organization.

^d^N/A: Not applicable.

**Search line.**

“(Musculoskeletal disease OR musculoskeletal disorder OR musculoskeletal pain OR chronic pain OR acute pain OR overuse pain OR chronic injury OR chronic injuries OR acute injury OR acute injuries OR overuse injury OR overuse injuries OR chronic disease OR acute disease OR overuse disease OR osteoporosis OR osteoarthritis OR rheumatoid arthritis OR tendinopathy OR tendinopathies OR rotator cuff OR lower extremity OR lower extremities OR upper extremity OR upper extremities OR hip OR knee OR foot OR hand OR ankle OR wrist OR elbow OR low back OR neck OR back OR spine OR shoulder OR arm OR leg OR muscle OR tendon OR ligament) AND (digital movement therapy OR digital movement therapies OR mobile health OR etherapy OR etherapies OR web-based intervention OR digital intervention OR computer-based intervention OR app-based intervention OR digital health application OR technology-assisted therapy OR technology-assisted therapies OR internet-based intervention OR computer-assisted therapy OR computer-assisted therapies OR health app OR mobile application OR smartphone OR mobile phone OR ehealth OR mhealth OR telerehabilitation OR telemedicine OR online intervention OR internet-delivered intervention) AND (osteopathy OR movement therapy OR movement therapies OR physical therapy OR physical therapies OR therapeutic exercise OR medical gymnastic OR traditional therapy OR traditional therapies OR manual therapy OR manual therapies OR physiotherapy OR no therapy OR no therapies OR conventional therapy OR conventional therapies OR no treatment OR no intervention OR watch-and-wait OR wait-and-see OR watch and wait OR wait and see) AND (randomized controlled trials).”

### Literature Search, Study Selection, and Risk of Bias

The literature search was performed on July 21, 2022, using the PubMed (MEDLINE), BISp (Federal Institute of Sport Science), Cochrane Library, and Web of Science databases. The search line included terms presented in Table 1. The “outcomes” category was not included in the search strategy but was considered in the subsequent study inclusion and selection process. No filters or other restrictions were used. The retrieved records were exported to a reference manager (EndNote 20, Clarivate). All duplicates were identified using the software and were removed after a manual review. On the basis of the defined eligibility criteria, studies were included or excluded by reviewing the titles, abstracts, and full texts. Full texts were accessed via public or open access and university accounts. If the full texts were not accessible, the authors were contacted. The study quality and the associated risk of bias were assessed using the Scottish Intercollegiate Guidelines Network checklist for randomized controlled trials [17]. The checklist consisted of 10 items related to the internal validity and 2 items related to the overall assessment of the studies. For each included study, all items were answered with “yes,” “no,” “can’t say,” or “not applicable.” The study quality was then finally rated throughout the “Scottish Intercollegiate Guidelines Network checklist for randomized controlled trials: Notes for completion of checklist” as “not acceptable,” “borderline,” “acceptable,” and “high,” as previously done [18]. The definitions of these quality classifications are presented in Textbox 2.

**Definitions for ratings of the overall methodological study quality.**

**High quality**

- Most criteria met. Little or no risk of bias. Results unlikely to be changed by further research.

**Acceptable quality**

- Most criteria met. Some flaws in the study with an associated risk of bias. Conclusions may change in the light of further studies.

**Borderline quality**

- Crude effect estimates have been presented or have been calculated (thus no confounders have been considered), but the study is otherwise acceptably sound with respect to other possible biases.

**Not acceptable quality**

- Either most criteria not met, or significant flaws relating to key aspects of study design. Conclusions likely to change in the light of further studies.

Note: Definitions according to Asker et al [18].

### Data Extraction and Synthesis of Results

Data extraction was performed according to the PICOS scheme. A best-evidence synthesis was conducted to clarify the evidence for digital physical health exercises on clinically relevant factors clustered as (1) localization of the musculoskeletal diseases, (2) patient-reported outcomes (according to the eligibility criteria), and (3) medical treatment types (conservative vs operative). Within these clusters, the study results were individually classified as positive, negative, or equal for each clinically relevant factor. In accordance with a previous study [14], the study results were classified as positive or negative if the intervention or control group showed statistically better significant study results than the other group for >50% of the outcome parameters that were used to examine the respective clinically relevant factors. If no statistically significant differences were reported between the intervention and control groups, the study results were classified as equal. With regard to the best-evidence synthesis, the established criteria [18] are summarized in Table 2, and to increase the validity, only studies with at least acceptable study quality were included [19].

**Table 2:** Criteria of best-evidence synthesis according to Asker et al [18].

Rating	Study quality	Criteria
Strong evidence	≥2 high-quality studies	≥75% consistent findings in these studies
Moderate evidence	1 high-quality study and/or ≥2 acceptable-quality studies	≥75% consistent findings in these studies
Limited evidence	1 acceptable-quality study and/or ≥1 borderline-quality study	N/A^a^
Conflicting evidence	Conflicting results in several studies of any quality	<75% of studies reported concordant results
No evidence	No admissible studies were found	N/A

^a^N/A: Not applicable.

## Results

### Literature Search, Study Selection, and Risk of Bias

**Figure 1:**
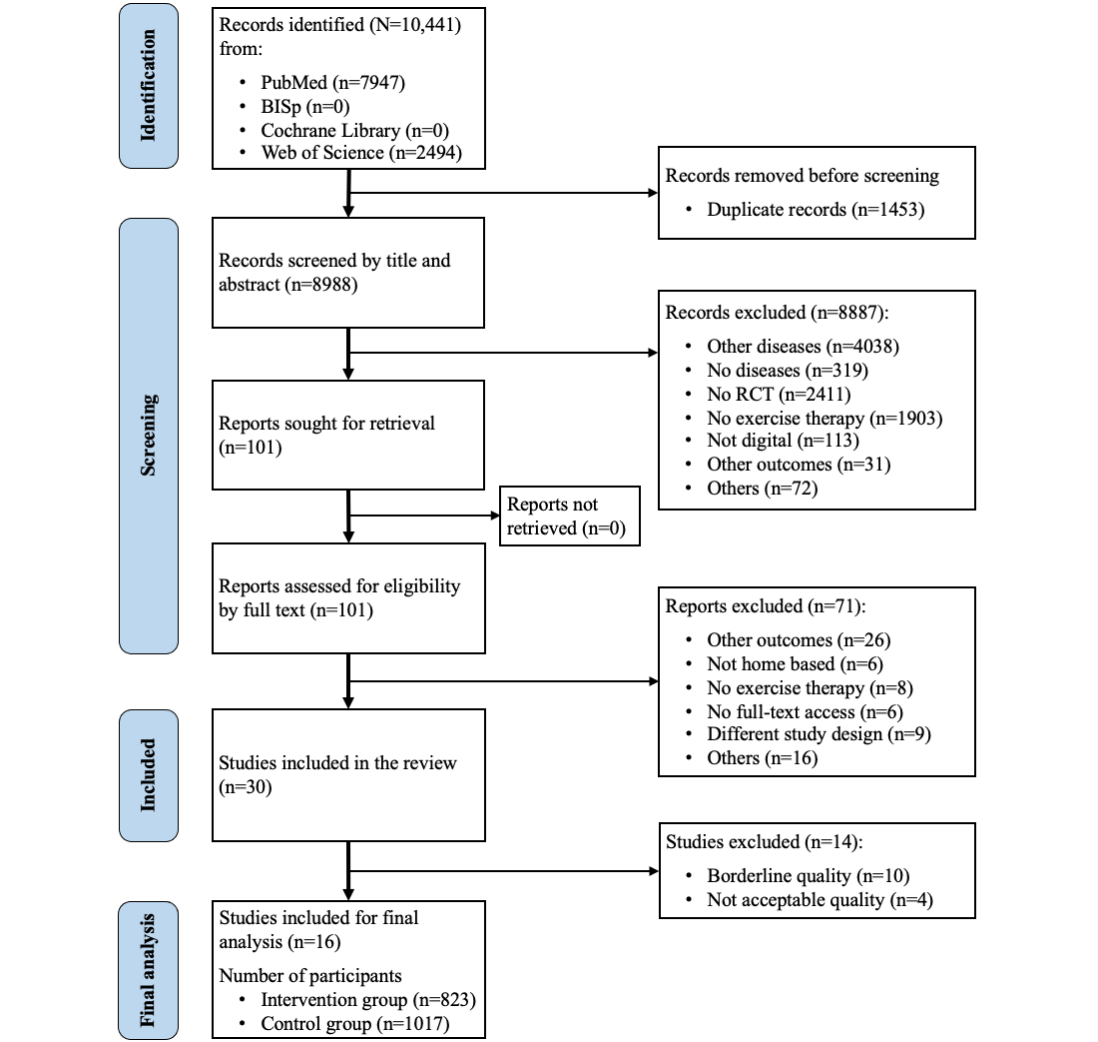
Flowchart of the literature search including the study selection process according to the PRISMA (Preferred Reporting Items for Systematic Reviews and Meta-Analyses) guidelines. RCT: randomized controlled trial.

Figure 1 shows the flowchart of the literature search including the study selection process according to the PRISMA guidelines. On the basis of the eligibility criteria, of 10,441 records, 30 (0.29%) studies were finally included in the risk of bias assessment. Although 1453 studies were identified as duplicates, 8958 studies that did not meet the inclusion criteria addressed no musculoskeletal diseases, were not randomized controlled trials, or addressed other outcomes. Table 3 summarizes the results of the risk of bias assessment.

There were 10 studies with high quality [20-29], 6 studies with acceptable quality [30-35], 10 studies with borderline quality [36-45], and 4 studies with not acceptable quality [46-49]. Thus, because of the not acceptable and borderline qualities of 14 studies, 16 studies were further analyzed and finally included in the best-evidence synthesis.

**Table 3:** Results of the 30 studies checked for the risk of bias assessment using the Scottish Intercollegiate Guidelines Network checklist.

Study	1.1	1.2	1.3	1.4	1.5	1.6	1.7	1.8 (%)	1.9	1.10	2.1	2.2	2.3	Total					Study quality
														Yes	No	CS^a^	N/A^b^		
Abadiyan et al [20]	Yes	Yes	Yes	No	Yes	Yes	Yes	3	CS	N/A	++^c^	Yes	Yes	8	1	1	1	High	
Allen et al [21]	Yes	Yes	Yes	No	Yes	Yes	Yes	13.1	Yes	N/A	++	Yes	Yes	9	1	0	1	High	
Blanquero et al [22]	Yes	Yes	Yes	No	Yes	Yes	Yes	0	Yes	N/A	++	Yes	Yes	9	1	0	1	High	
Choi et al [23]	Yes	Yes	Yes	CS	Yes	Yes	Yes	0	Yes	N/A	++	Yes	Yes	9	0	1	1	High	
Fatoye et al [24]	Yes	Yes	Yes	No	Yes	Yes	Yes	16	CS	N/A	++	Yes	Yes	8	1	1	1	High	
Fleischman et al [25]	Yes	Yes	Yes	No	Yes	Yes	Yes	16.6	Yes	N/A	++	Yes	Yes	9	1	0	1	High	
Moffet et al [26]	Yes	Yes	Yes	No	Yes	Yes	Yes	6.3	Yes	Yes	++	CS	Yes	9	1	1	0	High	
Nelligan et al [27]	Yes	Yes	Yes	Yes	Yes	Yes	Yes	12.6	Yes	Yes	++	Yes	Yes	11	0	0	0	High	
Nelson et al [28]	Yes	Yes	Yes	No	CS	Yes	Yes	1	Yes	N/A	++	Yes	Yes	8	1	1	1	High	
Özden et al [29]	Yes	Yes	Yes	Yes	Yes	Yes	Yes	7	CS	N/A	++	Yes	Yes	9	0	1	1	High	
Bennell et al [30]	Yes	Yes	Yes	No	Yes	Yes	Yes	10.1	Yes	N/A	+^d^	Yes	Yes	9	1	0	1	Acceptable	
Chhabra et al [31]	Yes	Yes	Yes	No	No	Yes	Yes	0	Yes	N/A	+	Yes	Yes	8	2	0	1	Acceptable	
Hardt et al [32]	Yes	Yes	Yes	No	Yes	CS	Yes	10	Yes	N/A	+	Yes	Yes	8	1	1	1	Acceptable	
Hernando-Garijo et al [33]	Yes	Yes	Yes	No	Yes	Yes	Yes	18	Yes	N/A	+	Yes	No	8	2	0	1	Acceptable	
Rodríguez Sánchez-Laulhé et al [34]	Yes	Yes	Yes	No	No	Yes	Yes	16	Yes	N/A	+	Yes	Yes	8	2	0	1	Acceptable	
Tousignant et al [35]	Yes	Yes	Yes	CS	Yes	Yes	Yes	15	CS	CS	+	Yes	Yes	8	0	3	0	Acceptable	
Anan et al [36]	Yes	Yes	Yes	No	Yes	CS	Yes	25.6	Yes	N/A	−^e^	CS	Yes	7	1	2	1	Borderline	
Bäcker et al [37]	Yes	Yes	Yes	No	Yes	Yes	Yes	42	No	N/A	−	Yes	Yes	8	2	0	1	Borderline	
Bossen et al [38]	Yes	Yes	Yes	No	Yes	Yes	Yes	24.6	Yes	N/A	−	Yes	Yes	9	1	0	1	Borderline	
Correia et al [39]	Yes	Yes	Yes	No	Yes	Yes	Yes	36	No	N/A	−	Yes	Yes	8	2	0	1	Borderline	
del Pozo-Cruz et al [40]	Yes	CS	CS	No	Yes	Yes	Yes	10	Yes	Yes	−	Yes	CS	7	1	3	0	Borderline	
Gohir et al [41]	Yes	Yes	Yes	No	Yes	Yes	Yes	28.1	Yes	N/A	−	Yes	Yes	9	1	0	1	Borderline	
Kloek et al [42]	Yes	Yes	Yes	No	Yes	Yes	Yes	35.1	Yes	CS	−	Yes	No	8	2	1	0	Borderline	
Piqueras et al [43]	Yes	Yes	Yes	No	No	Yes	Yes	26.5	CS	N/A	−	Yes	CS	6	2	2	1	Borderline	
Punt et al [44]	Yes	Yes	Yes	No	Yes	Yes	Yes	21	Yes	N/A	−	CS	Yes	8	1	1	1	Borderline	
Sandal et al [45]	Yes	Yes	Yes	No	Yes	Yes	Yes	23.6	Yes	CS	−	CS	Yes	8	1	2	0	Borderline	
Lara et al [46]	Yes	CS	CS	No	Yes	Yes	Yes	4	Yes	N/A	−−^f^	Yes	No	6	2	2	1	Not acceptable	
Lorig et al [47]	Yes	CS	CS	No	Yes	No	Yes	23.9	Yes	CS	−−	No	No	4	4	3	0	Not acceptable	
Shebib et al [48]	Yes	Yes	Yes	No	No	No	Yes	40.7	Yes	CS	−−	CS	Yes	6	3	2	0	Not acceptable	
Toelle et al [49]	Yes	No	No	No	Yes	Yes	Yes	14.9	No	N/A	−−	CS	CS	4	4	2	1	Not acceptable	

^a^CS: Cannot say.

^b^N/A: Not applicable.

^c^Low or no risk of bias.

^d^Associated risk of bias.

^e^Crucial risk of bias.

^f^High risk of bias.

### Study Characteristics

Table 4 presents the study characteristics of the 16 included studies according to the PICOS scheme.

The publication period ranged from 2011 [35] to 2022 [29,34], whereby 3 studies were published each in 2018 [21,31,32], 2019 [22,23,25], and 2021 [20,27,33]. The most common publication country was Australia, with 3 studies [27,28,30], followed by 2 studies each published by Spain [22,34], Canada [26,35], and the United States [21,25]. Across the 16 studies, 1840 patients were investigated, and the sample sizes ranged from 34 [33] to 350 patients [21]. The reported dropout rate was up to 18% (6/34) [33]. The average age of the patients varied from 38.5 [20] to 66 years [26,35], and the average female proportion across all studies reported was 61.35% (1008/1643) and varied from 51% (148/290) [25] to 100% (34/34) [33]. Regarding the localization of the musculoskeletal diseases, 7 studies were on knee-specific diseases such as total knee arthroplasty [25,26,32,35], knee osteoarthritis [21,27], and chronic knee pain [30]. This was followed by 4 studies on back-specific diseases such as low back pain [24,29,31] and chronic neck pain [20]. A total of 2 studies were on hand-specific diseases [22,34], whereas only 1 study was found for each full body [33], shoulder-specific diseases [23], and hip-specific diseases [28]. Regarding the patient-reported outcomes, 14, 12, 6, and 5 studies investigated pain [20-23,25-27,29-35], function [21,23,25-30,32-35], disability [20,22,24,29,31,34], and quality of life [20,27-30], respectively. In the 16 included studies, 26 different patient-reported outcomes were investigated. With regard to the digital health interventions, 7, 5, and 4 studies used app-based [20,22,23,28,31,32,34], web-based [21,25,27,29,30], and telerehabilitation-based physical exercises [24,26,33,35], respectively, whereby the duration of the digital health interventions ranged from 7 days [32] to 12 months [21]. As control groups, 9 studies used physiotherapy [20,21,23,25,26,28,31,32,35]; 4 studies used paper-based exercises [22,25,29,34]; 2 studies used internet-based information material [27,30]; and 1 study each used global postural re-education [20], waiting list [21], clinic-based McKenzie therapy [24], and no therapy [33].

**Table 4:** Summary table of all study characteristics according to the population, intervention, comparison, outcome, study design (PICOS) scheme.

Study	Population and setting	Intervention and assessment	Outcomes
Abadiyan et al [20]	Sample size: n=60 Average age: 38.5 y Female: 55% Disease: chronic neck pain Country: Iran	I^a^ (“Seeb” app+ GPR^b^): n=20 C1^c^ (GPR alone): n=20 C2^d^ (conventional PT^e^) n=20 Duration: 8 wk Survey dates: baseline, 8 wk	Drop out: 3% I: 5%, C1: 5%, and C2: 0% Pain: app+GPR>GPR app+GPR>PT Neck disability index: app+GPR>GPR app+GPR>PT GPR>PT Quality of life: app+GPR>PT GPR>PT
Allen et al [21]	Sample size: n=350 Average age: 65.3 y Female: 71.7% Disease: knee osteoarthritis Country: United States	I (IBET^f^): n=142 C1 (conventional PT): n=140 C2 (WL^g^): n=68 Duration: 12 mo Survey dates: baseline, 4, and 12 mo	Drop out: 13.1% I: 21.1%, C1: 7.9%, and C2: 7% Western Ontario and McMaster Universities Osteoarthritis Index (WOMAC^h^) and other functional tests IBET=PT=WL
Bennell et al [30]	Sample size: n=148 Average age: 61.2 y Female: 56.1% Disease: chronic knee pain Country: Australia	I: internet-based education material supported by videoconferences with physiotherapist for home exercises (n=74) Control group: internet-based education material only (n=74) Duration: 9 mo Survey dates: baseline, 3, and 9 mo	Drop out: 10.1% I: 11% and C: 10% Pain and function: education+PT>education Quality of life: education+PT>education
Blanquero et al [22]	Sample size: n=50 Average age: 50.0 y Female: 82% Disease: carpal tunnel release Country: Spain	I: ReHand app for physical home training (n=25) Control group: paper and home-based physical exercise program (n=25) Duration: 4 wk Survey dates: baseline, 4 wk	Drop out: 0% Hand disability and pain: app based>paper based
Chhabra et al [31]	Sample size: n=93 Average age: 41.2 y Female: not reported Disease: chronic low back pain Country: India	I: Snapcare app for physical home training (n=45) Control group: conventional therapy (n=48) Duration: 12 wk Survey dates: baseline, 12 wk	Drop out: 0% Pain: app based=conventional Disability: app based>conventional Current Symptom Score: app based>conventional
Choi et al [23]	Sample size: n=84 Average age: 54.5 y Female: 68% Disease: frozen shoulder Country: Korea	I: app (no name given) for physical home training (n=42) Control group: conventional home-based self-exercises (n=42) Duration: 3 mo Survey dates: baseline, 4, 8, and 12 wk	Drop out: 0% Pain and range of motion: app based=conventional
Fatoye et al [24]	Sample size: n=56 Average age: 48.7 y Female: not reported Disease: chronic low back pain Country: Nigeria	I: telerehabilitation home-based McKenzie therapy (TBMT^i^; n=24) Control group: clinic-based McKenzie therapy (CBMT^j^; n=32) Duration: 8 wk Survey dates: baseline, 4, and 8 wk	Drop out: 16% I: 13% and C: 19% Disability: TBMT=CBMT
Fleischman et al [25]	Sample size: n=290 Average age: 65.0 y Female: 51% Disease: total knee arthroplasty Country: United States	I: web-based PT at home (n=96) C1: paper-based PT at home ( n=97) C2: formal outpatient PT (n=97) Duration: 6 mo Survey dates: baseline, 4-6 wk, 6 mo	Drop out: 15.9% I: 17%, C1: 27%, and C2: 6% Knee flexion and Knee Injury and Osteoarthritis Outcome Score (KOOS^k^): Web PT=paper PT=PT
Hardt et al [32]	Sample size: n=60 Average age: 65.9 y Female: 57% Disease: total knee arthroplasty Country: Germany	I: PT+“GenuSport” app (PT+app; n=33) Control group: PT (n=27) Duration: 7 d Survey dates: daily for 7 d	Drop out: 10% I: 15% and C: 7% Active range of motion, pain, function, KOOS, and Knee Society Score: PT+app>PT
Hernando-Garijo et al [33]	Sample size: n=34 Average age: 53.4 y Female: 100% Disease: fibromyalgia Country: Mexico	I: telerehabilitation with home-based aerobic exercises (n=17) Control group: no additional intervention (n=17) Duration: 15 wk Survey dates: baseline, 15 wk	Drop out: 18% I: 18% and C: 18% Pain: telerehabilitation>nothing Physical function: telerehabilitation=nothing
Moffet et al [26]	Sample size: n=205 Average age: 66.0 y Female: 51.2% Disease: total knee arthroplasty Country: Canada	I: home-based telerehabilitation (n=104) Control group: home-visiting PT (n=101) Duration: 2 mo Survey dates: baseline, 2, and 4 mo	Drop out: 6.3% I: 9.6% and C: 2.9% WOMAC, KOOS, function, and range of motion: telerehabilitation=PT
Nelligan et al [27]	Sample size: n=206 Average age: 60.0 y Female: 61.2% Disease: knee osteoarthritis Country: Australia	I: website (information+active exercises) and text messages (n=103) Control group: website with information only (n=103) Duration: 24 wk Survey dates: baseline, 24 wk	Drop out: 12.6% I: 12.6% and C: 12.6% Pain, WOMAC, KOOS, quality of life: website information+exercise>website information only
Nelson et al [28]	Sample size: n=70 Average age: 64.5 y Female: 63% Disease: total hip replacement Country: Australia	I: telerehabilitation and technology-based home exercise (n=35) Control group: PT and paper-based home exercise (n=35) Duration: 6 wk Survey dates: baseline, 6 wk, 6 mo	Drop out: 1% I: 3% and C: 0% Quality of life and function: telerehabilation+exercise=PT+exercise
Özden et al [29]	Sample size: n=50 Average age: 41.3 y Female: 60% Disease: low back pain Country: turkey	I: telerehabilitation with Fizyoweb software (n=25) Control group: same exercises with paper-based instructions (n=25) Duration: 8 wk Survey dates: baseline, 8 wk	Drop out: 7% I: 7% and C: 7% Pain, function, disability, and quality of life: telerehabilation>paper based
Rodríguez Sánchez-Laulhé et al [34]	Sample size: n=36 Average age: 59.8 y Female: 61% Disease: rheumatoid arthritis Country: Spain	I: CareHand app for exercises and self-management and monitoring tools (n=14) Control group: paper-based home exercises (n=22) Duration: 3 mo Survey dates: baseline, 1, 3, and 6 mo	Drop out: 16% I: 7% and C: 22% Function: app based>paper based Pain and disability for upper extremity: app based=paper based
Tousignant et al [35]	Sample size: n=48 Average age: 66.0 y Female: not reported Disease: total knee arthroplasty Country: Canada	I: telerehabilitation by videoconference with a physiotherapist (n=24) Control group: conventional PT (n=24) Duration: 2 mo Survey dates: baseline, 2, and 6 mo	Drop out: 15% I: 12% and C: 17% Disability: telerehabilation=conventional PT Function: telerehabilation>conventional PT Functional activity, physical functioning, and physical pain: conventional PT>telerehabilation

^a^I: Intervention group.

^b^GPR: Global postural re-education.

^c^C1: Control group 1.

^d^C2: Control group 2.

^e^PT: Physiotherapy.

^f^IBET: Internet-based exercise training.

^g^WL: Waitlist.

^h^WOMAC: Western Ontario and McMaster Universities Osteoarthritis Index.

^i^TBMT: Telerehabilitation home-based McKenzie therapy.

^j^CBMT: Clinic-based McKenzie therapy.

^k^KOOS: Knee Injury and Osteoarthritis Outcome Score.

### Synthesis of Results by Best‐Evidence Synthesis

Tables 5, 6, and 7 show the results of the best-evidence synthesis with regard to the cluster of the localization of the musculoskeletal diseases, patient-reported outcomes, and medical treatment types, respectively.

**Figure 2:**
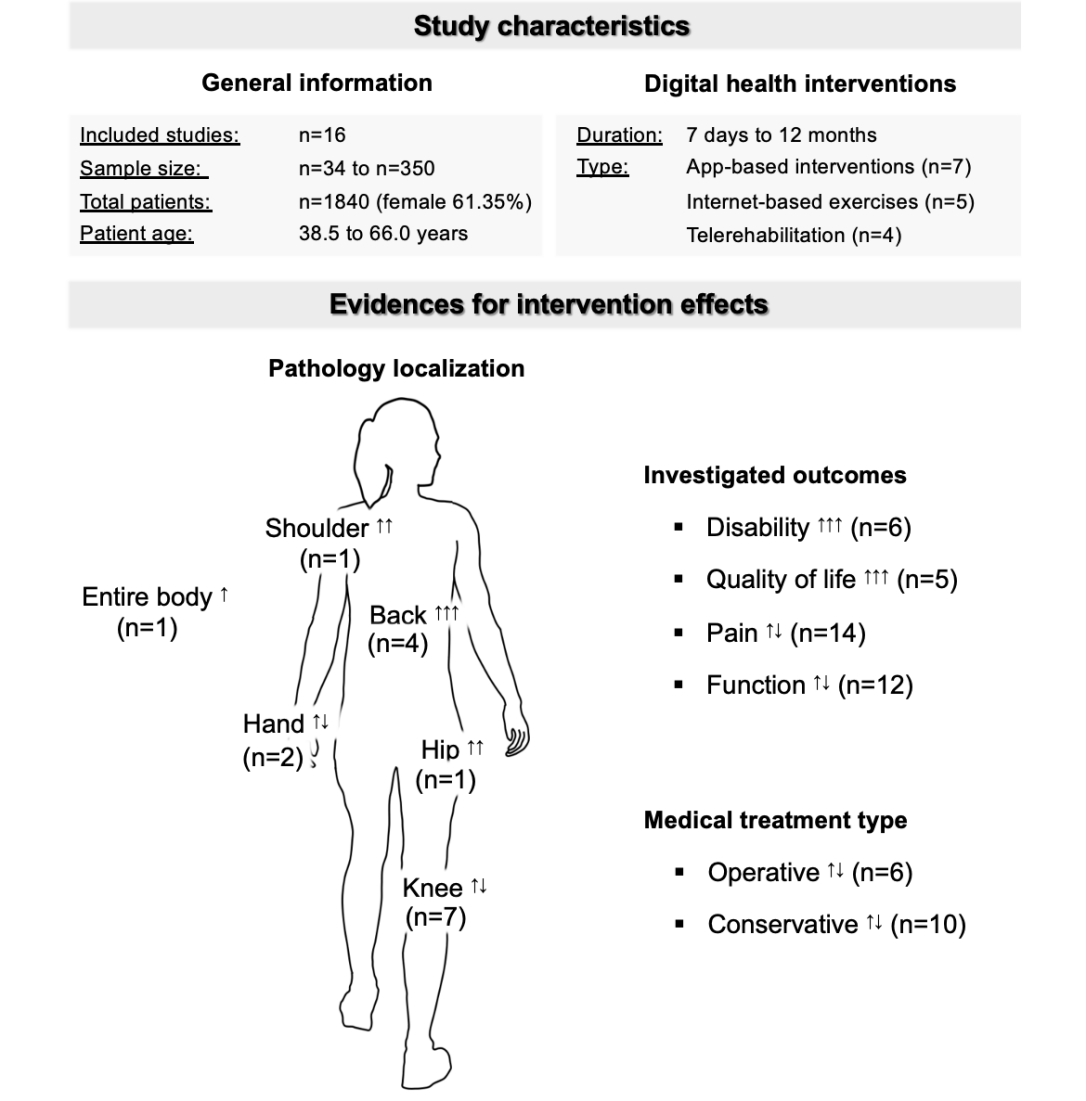
Visualization of the evidence found across the 3 defined clusters for studies included in the best-evidence synthesis. ↑↑↑: strong evidence, ↑↑: moderate evidence, ↑: limited evidence, and ↑↓: conflicting evidence.

Regarding the localization of the musculoskeletal diseases, there was strong evidence that digital physical health exercises had a positive impact on the musculoskeletal diseases located in the back. Although moderate evidence was obtained for diseases located in the shoulder and hip, evidence for fibromyalgia (the entire body) is limited. Conflicting evidence was found for diseases located in the knee and hand. For the patient-reported outcomes, there was strong evidence that digital physical health exercises had a positive impact on disability and quality of life. Conflicting evidence was found for pain and function. Regarding the medical treatment types, operative and conservative therapies both achieved conflicting evidence. Figure 2 shows the evidence found across the 3 defined clusters for studies included in the best-evidence synthesis.

**Table 5:** Best-evidence synthesis for the localization of the musculoskeletal diseases.

Localization	Study	Musculoskeletal disease	Results	Study quality	Evidence
Back	Abadiyan et al [20]	Chronic neck pain	+^a^	High	Strong^b^
Back	Chhabra et al [31]	Chronic low back pain	+	Acceptable	Strong^b^
Back	Fatoye et al [24]	Chronic low back pain	=^c^	High	Strong^b^
Back	Özden et al [29]	Chronic low back pain	+	High	Strong^b^
Shoulder	Choi et al [23]	Frozen shoulder	=	High	Moderate
Hip	Nelson et al [28]	Total hip arthroplasty	=	High	Moderate
Full body	Hernando-Garijo et al [33]	Fibromyalgia	=	Acceptable	Limited
Knee	Allen et al [21]	Knee osteoarthritis	=	High	Conflicting^b^
Knee	Bennell et al [30]	Chronic knee pain	+	Acceptable	Conflicting^b^
Knee	Fleischman et al [25]	Total knee arthroplasty	=	High	Conflicting^b^
Knee	Hardt et al [32]	Total knee arthroplasty	+	Acceptable	Conflicting^b^
Knee	Moffet et al [26]	Total knee arthroplasty	=	High	Conflicting^b^
Knee	Nelligan et al [27]	Knee osteoarthritis	+	High	Conflicting^b^
Knee	Tousignant et al [35]	Total knee arthroplasty	+	Acceptable	Conflicting^b^
Hand	Blanquero et al [22]	Carpal tunnel release	+	High	Conflicting^b^
Hand	Rodríguez Sánchez−Laulhéet al [34]	Rheumatoid arthritis	=	Acceptable	Conflicting^b^

^a^>50% of the outcomes were significantly better in the intervention group than in the control group.

^b^The level of evidence was determined from all studies in the same localization.

^c^No statistically significant difference between the intervention and control groups.

**Table 6:** Best-evidence synthesis for the patient-reported outcomes of the musculoskeletal diseases.

Outcomes	Study	Assessment tools	Results	Study quality	Evidence
Disability	Abadiyan et al [20]	Neck Disability Index	+^a^	High	Strong^b^
Disability	Blanquero et al [22]	Disabilities of Arm, Shoulder and Hand Questionnaire	+	High	Strong^b^
Disability	Chhabra et al [31]	Modified Oswestry Disability Index	+	Acceptable	Strong^b^
Disability	Fatoye et al [24]	Oswestry Disability Index	=^c^	High	Strong^b^
Disability	Özden et al [29]	Oswestry Disability Index	+	High	Strong^b^
Disability	Rodríguez Sánchez-Laulhé et al [34]	Disabilities of Arm, Shoulder and Hand Questionnaire	=	Acceptable	Strong^b^
Quality of life	Abadiyan et al [20]	Short Form Health 36 Questionnaire	+	High	Strong^b^
Quality of life	Bennell et al [30]	Assessment of Quality of Life−2	+	Acceptable	Strong^b^
Quality of life	Nelligan et al [27]	Assessment of Quality of Life-6D	+	High	Strong^b^
Quality of life	Nelson et al [28]	Short Form Health 12 Questionnaire/European Quality of Life 5 Dimensions 5 Level Version	=	High	Strong^b^
Quality of life	Özden et al [29]	Short Form Health 36 Questionnaire	+	High	Strong^b^
Pain	Abadiyan et al [20]	Visual analog scale	+	High	Conflicting^b^
Pain	Allen et al [21]	WOMAC^d^	=	High	Conflicting^b^
Pain	Bennell et al [30]	Numeric rating scale	+	Acceptable	Conflicting^b^
Pain	Blanquero et al [22]	Visual analog scale	+	High	Conflicting^b^
Pain	Chhabra et al [31]	Numeric rating scale, Current Symptom Score	=	Acceptable	Conflicting^b^
Pain	Choi et al [23]	Visual analog scale	=	High	Conflicting^b^
Pain	Fleischman et al [25]	KOOS^e^	=	High	Conflicting^b^
Pain	Hardt et al [32]	Numeric rating scale	+	Acceptable	Conflicting^b^
Pain	Hernando-Garijo et al [33]	Visual analog scale	+	Acceptable	Conflicting^b^
Pain	Moffet et al [26]	WOMAC	=	High	Conflicting^b^
Pain	Nelligan et al [27]	Numeric rating scale	+	High	Conflicting^b^
Pain	Özden et al [29]	Visual analog scale	+	High	Conflicting^b^
Pain	Rodríguez Sánchez-Laulhé et al [34]	Visual analog scale	=	Acceptable	Conflicting^b^
Pain	Tousignant et al [35]	WOMAC	−^f^	Acceptable	Conflicting^b^
Function	Allen et al [21]	WOMAC/30-s chair stand test/Timed up and go test/2-min step test, single-leg stand	=	High	Conflicting^b^
Function	Bennell et al [30]	WOMAC	+	Acceptable	Conflicting^b^
Function	Choi et al [23]	Range of motion	=	High	Conflicting^b^
Function	Fleischman et al [25]	KOOS	=	High	Conflicting^b^
Function	Hardt et al [32]	Range of motion/Timed up and go test/10-m walk test/30-s chair stand test/Knee Society Score	+	Acceptable	Conflicting^b^
Function	Hernando-Garijo et al [33]	Arm curl test, 6-min walk test	=	Acceptable	Conflicting^b^
Function	Moffet et al [26]	KOOS/Stair test/6-min walk test	=	High	Conflicting^b^
Function	Nelligan et al [27]	WOMAC, KOOS	+	High	Conflicting^b^
Function	Nelson et al [28]	Timed up and go test	=	High	Conflicting^b^
Function	Özden et al [29]	Timed up and go test	+	High	Conflicting^b^
Function	Rodríguez Sánchez-Laulhé et al [34]	Michigan Hand Outcome Questionnaire	+	Acceptable	Conflicting^b^
Function	Tousignant et al [35]	WOMAC/Timed up and go test/Functional Autonomy Measurement System	−	Acceptable	Conflicting^b^

^a^>50% of the outcomes were significantly better in the intervention group than in the control group.

^b^The level of evidence was determined from all studies in the same outcomes.

^c^No statistically significant differences between the intervention and control groups.

^d^WOMAC: Western Ontario and McMaster Universities Osteoarthritis Index.

^e^KOOS: Knee Injury and Osteoarthritis Outcome Score.

^f^>50% of the outcomes were significantly better in the control group than in the intervention group.

**Table 7:** Best-evidence synthesis for the medical treatment types.

Therapy	Study	Musculoskeletal disease	Results	Study quality	Evidence
Operative	Blanquero et al [22]	Carpal tunnel release	+^a^	High	Conflicting^b^
Operative	Fleischman et al [25]	Total knee arthroplasty	=^c^	High	Conflicting^b^
Operative	Hardt et al [32]	Total knee arthroplasty	+	Acceptable	Conflicting^b^
Operative	Moffet et al [26]	Total knee arthroplasty	=	High	Conflicting^b^
Operative	Nelson et al [28]	Total hip arthroplasty	=	High	Conflicting^b^
Operative	Tousignant et al [35]	Total knee arthroplasty	+	Acceptable	Conflicting^b^
Conservative	Abadiyan et al [20]	Chronic neck pain	+	High	Conflicting^b^
Conservative	Allen et al [21]	Knee osteoarthritis	=	High	Conflicting^b^
Conservative	Bennell et al [30]	Chronic knee pain	+	Acceptable	Conflicting^b^
Conservative	Chhabra et al [31]	Chronic low back pain	+	Acceptable	Conflicting^b^
Conservative	Choi et al [23]	Frozen shoulder	=	High	Conflicting^b^
Conservative	Fatoye et al [24]	Chronic low back pain	=	High	Conflicting^b^
Conservative	Hernando-Garijo et al [33]	Fibromyalgia	=	Acceptable	Conflicting^b^
Conservative	Nelligan et al [27]	Knee osteoarthritis	+	High	Conflicting^b^
Conservative	Özden et al [29]	Chronic low back pain	+	High	Conflicting^b^
Conservative	Rodríguez Sánchez-Laulhé et al [34]	Rheumatoid Arthritis	=	Acceptable	Conflicting^b^

^a^>50% of the outcomes were significantly better in the intervention group than in the control group.

^b^The level of evidence was determined from all studies in the same therapy.

^c^No statistically significant difference between the intervention and control groups.

## Discussion

### Principal Findings

This systematic review aimed to evaluate the impact of digital physical health exercises on patients with musculoskeletal diseases concerning the localization of the musculoskeletal disease, patient-reported outcomes, and medical treatment types. In addition, a best-evidence synthesis was conducted to estimate the direction and strength of the existing evidence. The main findings were that (1) strong evidence was found for a positive impact on musculoskeletal diseases located in the back and on the patient-reported outcomes of disability and quality of life and (2) moderate evidence was found for a positive impact on musculoskeletal diseases located in the shoulder and hip.

The first main finding was that strong evidence was found for a positive impact on musculoskeletal diseases located in the back and on the patient-reported outcomes of disability and quality of life (Figure 2). Our findings are partly supported by a previous systematic review with a meta-analysis [13] showing moderate-quality evidence for the positive impact on the patient-reported outcome of disability. In contrast to the previous review [13] and to another systematic review [12], conflicting evidence for the patient-reported outcomes of pain and function was found. It should be noted that 1 study [12] found some clinical benefits for pain and function but did not conduct an evidence synthesis or a meta-analysis. In addition, the outcomes of pain and function represent health-related outcomes, and the outcomes of disability and quality of life are the resulting consequences. Therefore, pain acts as a protective mechanism and can lead to disability [50]. With appropriate exercises, patients learn to compensate for their disabilities [20,30], whereas exercise alone can provoke pain [51]. As disabilities are part of the concept of health-related quality of life [52], these outcomes are mutually dependent, and identical strong evidence is plausible.

In addition, it should be mentioned that both previous systematic reviews included all types of digital health interventions, and we explicitly focused our systematic review on the impact of digital physical health exercises. Regarding this, our findings add that this type of intervention shows strong evidence to have an overall positive impact on the musculoskeletal diseases located in the back, independent of the investigated outcomes [20,24,29,31]. Back-related musculoskeletal diseases usually arise because of muscular causes and are often caused by a lack of physical activity [53]. Participants recruited in back pain–related studies are often middle-aged and have an office occupation [20]. The use of digital physical health exercises in such patients can be considered highly effective because of the increased physical activity targeting muscle strengthening and the teaching of exercise techniques [20,24,29,31]. Overall, the application of digital physical health exercise in patients with musculoskeletal diseases shows versatile positive effects, especially for musculoskeletal diseases located in the back and for the improvement of disabilities and quality of life. However, the type of digital health interventions seems to influence the effects on the specific patient-reported outcome, and more studies to investigate this relationship are needed.

The second main finding was that moderate evidence was found for a beneficial effect on musculoskeletal diseases of the shoulder and hip (Figure 2). As this systematic review is the first to evaluate the association between digital physical health exercises and different localizations of musculoskeletal diseases, no evidence levels from previous research is available for clarification. There is only 1 other systematic review on the effectiveness of digital health interventions for total hip arthroplasty [54]. The review found no significant improvements in the studied patient-reported outcomes. For the shoulder, another systematic review examined the effectiveness of telerehabilitation for musculoskeletal diseases compared with normal in-person physiotherapy [55] and found very low to low evidence. In this context, our findings suggest that digital physical health exercises may also be effective in treating musculoskeletal diseases of the shoulder and hip. However, it should be noted that only 1 study each was found for shoulder- and hip-specific musculoskeletal diseases, whereas several studies were found for back- or knee-specific musculoskeletal diseases (Table 5). Therefore, our results must be interpreted with caution, as a small number of high-quality studies may result in stronger evidence, according to the definitions of the best-evidence synthesis [18], than the presence of many lower-quality studies. Overall, the results demonstrated that digital physical health exercises could have a positive effect on a variety of health-related outcomes, regardless of the localization of the musculoskeletal diseases. However, the number of studies investigating the relationship between the effectiveness of digital health interventions and the localization of musculoskeletal diseases is small, and more studies are needed, especially for localizations other than the knee and back.

An additional interesting finding is the conflicting evidence in the medical treatment types concerning operative and conservative approaches (Figure 2). Although the underlying reasons remain unknown, it can be stated that the operative treatment (ie, carpal tunnel release and total knee arthroplasty) has no impact on the overall stimulus-response mechanism of the digital intervention, requiring further studies for clarification.

### Limitations

Although this systematic review increases knowledge of the positive impacts of digital physical health exercises on musculoskeletal diseases, there are a few limitations. Because of the heterogeneity of the included studies (eg, different numbers of patients, interventions, body regions, and control groups), a meta-analysis could not be performed. Instead, and as an established alternative approach, a best-evidence synthesis [18] was used. A strength of this approach is that it is possible to estimate an evidence level for various categories despite the large study heterogeneity. However, a limitation is that no quantitative analysis (eg, in terms of statistical significance) can be conducted [56]. An additional limitation of our review is that we did not register the study plan in PROSPERO a priori. The reason is that according to the PRISMA guidelines, registration is currently recommended but not mandatory [15]. Furthermore, all studies that included digital health interventions beyond active exercises were not included. Therefore, some studies could be lost, but the aspect of physical exercise as an established clinical treatment for musculoskeletal diseases could be focused on for the first time.

### Conclusions

There is strong to moderate evidence for the beneficial impact of digital physical health exercises for musculoskeletal diseases located in the back, shoulder, and hip. There is limited or conflicting evidence for other localizations. In addition, strong evidence was found for the patient-reported outcomes of disability and quality of life, whereas conflicting evidence exists for other commonly investigated patient-reported outcomes such as pain and function. Thus, digital physical health exercises could have a positive effect on a variety of health-related outcomes of musculoskeletal diseases. To implement digital physical health exercises in evidence-based medicine for musculoskeletal diseases, more high-quality randomized controlled trials are needed to clarify the relationship between the impact of digital physical health exercises and clinically relevant factors such as localization, patient-reported outcomes, and medical treatment types.

## Appendix

Multimedia Appendix 1 PRISMA 2020 Checklist.

## Abbreviations

PICOS: Population Intervention Comparison Outcome Study design
PRISMA: Preferred Reporting Items for Systematic Reviews and Meta-Analyses
